# Isolation and genomic characterization of one novel goose astrovirus causing acute gosling gout in China

**DOI:** 10.1038/s41598-023-37784-9

**Published:** 2023-06-29

**Authors:** Zhifeng Peng, Dongsheng Gao, Xinghui Song, Huimin Huang, Xiaozhan Zhang, Zenghai Jiang, Hongxing Qiao, Chuanzhou Bian

**Affiliations:** 1 College of Veterinary Medicine, Henan University of Animal Husbandry and Economy, No. 6 Longzihu North Road, Zhengzhou, 450046 Henan China; 2 Henan Dahenong Animal Husbandry Co. Ltd., Zhengzhou, 450000 China

**Keywords:** Evolution, Genetics, Microbiology

## Abstract

Novel goose astrovirus (NGAstV) is a member of the genus *Avain Avastrovirus* (AAstV) and the family *Astroviridae*. NGAstV-associated gout disease has caused huge economic losses to the goose industry worldwide. Since early 2020, NGAstV infections characterized by articular and visceral gout emerged continuously in China. Herein, we isolated a GAstV strain from goslings with fatal gout disease and sequenced its complete genome nucleotide sequence. Then we conducted systematic genetic diversity and evolutionary analysis. The results demonstrated that two genotypic species of GAstV (GAstV-I and GAstV-II) were circulating in China, and GAstV-II sub-genotype IId had become the dominant one. Multiple alignments of amino acid sequences of GAstV capsid protein revealed that several characteristic mutations (E456D, A464N, and L540Q) in GAstV-II d strains, as well as additional residues in the newly identified isolate which varied over time. These findings enrich the understanding of the genetic diversity and evolution of GAstV and may facilitate the development of effective preventive strategies.

## Introduction

NGAstV is a member of the genus AAstV and the family *Astroviridae*. It is a non-enveloped, positive-sense and single-stranded RNA virus with genome of 6.8 to 7.9 kb in size. The genome of NGAstVs consists of a 5′-untranslated region (UTR), three open reading frames (ORFs 1a, 1b, and 2), a 3′-UTR as well as a poly (A) tail^1^. ORF1a encodes a 3C-like serine protease motif, ORF1b encodes an RNA-dependent RNA polymerase (RdRP), while ORF2 encodes capsid protein that is required for virion formation^2^. Based on host spectrum, the family *Astroviridae* is divided into the genus *Mammalian astroviruses* (MAstV) and the genus AAstV^3^. The genus AAstV consist of three species: *Avastrovirus* 1, which includes turkey astrovirus 1 (TAstV-1), and is associated with turkeys′ diarrhea. *Avastrovirus* 2, which includes avian nephritis virus 1 (ANV-1) and avian nephritis 2 (ANV-2), can cause nephritis, mild growth retardation and hatchery disease in infected chickens and turkeys. *Avastrovirus* 3 is comprised of turkey astrovirus 2 (TAstV-2), and duck astrovirus 1 (DAstV-1) and 2 (DAstV-2). Two genotypic species of GAstV are currently known. GAstV-I is represented by FLX (KY271027)^4^, while GAstV-II is represented by HN1G (KY807085) and is designated NGAstV^5^. Phylogenetic analysis showed that both genotype species are highly divergent^6^.

Since 2017, the Chinese goose industry has experienced a severe outbreak of fatal visceral gout in goslings of 5–20 days old. The common feature at post-mortem of this disease is visceral urate deposition on the surfaces of heart, liver, kidneys, and as well as in joints^6–9^. Several studies have confirmed the causative agent was NGAstV^6,10,11^. This virus has caused considerable economic losses to goose industry worldwide^8–10,12,13^. In addition, the vertical transmission of NGAstVs poses greater challenges to prevention and control the disease^14^. However, there is currently no effective preventive strategy to protect against this virus.

A sufficient understanding of the genetic variation of NGAstVs is the basis for the development of preventive strategies to protect from this virus. Herein, we isolated a NGAstV strain from diseased goslings with acute urate deposition on the surfaces of viscera in Henan province, China. To better understand the molecular characteristics of the NGAstV, the complete genome of the newly identified NGAstV strain was cloned, sequenced, and its phylogeny and mutations were analyzed. Meanwhile, this study systematically described the genetic and evolutionary characteristics of the ongoing NGAstV strains. The findings enrich the available molecular information on NGAstV and facilitate the development of proper control programs.

## Material and methods

### Case history and samples

In December 2021, a fatal epidemic characterized by visceral gout of goslings emerged on a goose farm in Zhengzhou, Henan province. The disease started in 1–2 weeks old goslings with mortality rate of over 30% on the goose farm. The diseased goslings exhibited lameness, white diarrhea, and anorexia. Post-mortem examination revealed urate deposition on the surfaces of viscera, including heart, liver, and kidneys. Thirteen diseased goslings selected randomly were send to the Veterinary Diagnostic Laboratory, Henan University of Animal Husbandry and Economy, for diagnosis.

### Virus detection

To determine the causative agents of the disease, several potential viruses were detected, including goose astrovirus (GAstV), tembusu virus (TMUV), goose parvovirus (GPV), goose reovirus (GRV), and goose haemorrhagic polyomavirus (GHPV), avian influenza virus (AIV), and avian paramyxovirus-1 (APMV-1). The samples from two gosling selected randomly were pooled, and processed by extracting DNA/RNA using a Takara Viral DNA/RNA extraction Kit (Takara, Dalian, China). Subsequently, GRV, TMUV, GPV, GHPV, APMV-1 and GAstV were detected using PCR/RT-PCR, with specific primers as described previously (Supplementary Table S1)^8,15–18^. Subsequently, the samples from 13 goslings were detected for GAstV retrospectively.

### Virus isolation and quantitation

The GAstV-positive samples of liver from the diseased goslings were homogenized in phosphate-buffered saline (PBS, pH 7.2), freeze-thawed three times, and centrifuged at 8000×*g* for 10 min. The supernatants were filtered through 0.22 μm filter (Millipore). Subsequently, 0.2 mL supernatant was inoculated into the allantoic cavity of 15-day-old healthy goose embryos. The embryos were incubated in a 37 ℃ incubator, and monitored daily. If the embryos died 3–4 days post-inoculation, the dead embryos were collected sterilely for the observation of lesions, the allantoic fluids were then harvested for another round of inoculation. At the fourth passage, the allantoic fluids and goose embryos were harvested sterilely and stored at − 80 °C. The viral DNA/RNA extracted from the allantoic fluids were used to detect the potential causative agents, including GRV, TMUV, GPV, GHPV, APMV-1 and GAstV.

To determine the infectious titers of the P5 and P10 passage, the virus suspensions were inoculated into 25-cm^2^ culture flasks respectively, and the supernatants at 48 h post-inoculation were collected to tissue culture infective dose 50% (TCID_50_) determination. Goose embryo fibroblast (GEF) cells were seeded in 96-well plates (Supplementary Figure S1). When reached 60% confluence, the cells were washed three times with PBS buffer. The virus solution was diluted (10^−1^–10^−9^) with DMEM. Virus dilutions were then added to the cells with six replicates of 100 μL/well per dilution. Normal control cells were treated with 200 μL of 2% DMEM maintenance medium. After incubation in a 5% CO_2_, incubator at 37 ℃. The TCID_50_ of the P5 and P10 passage virus was calculated using the Reed–Muench method^19^.

### Determination of complete genome

To determine the complete genome nucleotide sequence of the newly isolated NGAstV strain, total RNA was extracted from the clinical samples and goose embryo allantoic fluids using a Takara Viral DNA/RNA extraction kit (Takara, Dalian, China). Viral genomic fragments were amplified using overlap PCR with primers as previously reported (Supplementary Table S2)^6,8^. The 5′-UTR and 3′-UTR of the genome were amplified by 5′ and 3′ rapid amplification of cDNA ends (RACE) strategies as previously reported^8^. The PCR amplicons were gel-purified and cloned into pMD18-T vector (Takara, Dalian, China) for sequencing.

### Phylogenetic analysis and sequence comparison

Isolated sequence similarity searches were performed with BLAST in the NCBI database (https://blast.ncbi.nlm.nih.gov/Blast.cgi). Based on complete genome nucleotide sequences and deduced amino acid sequences of ORF2, the phylogenetic trees were conducted by MEGA 6.0 software using the neighbor-joining method with bootstrap value of 1000, respectively. Subsequently, using DNAStar 7.0 software, the nucleotide and deduced amino acid sequences of the newly identified NGAstV HNZZ-4 were compared with the known sequences of GAstVs in GenBank (Supplementary Table S3), and the amino acid mutation sites were further analyzed.

### Ethics approval

All experiments involving animals were performed based on the guidelines for the care and use of animals and approved by the Animal Ethics Committee of Henan University of Animal Husbandry and Economy (HNUAHE ER2023-015), all methods were carried out in accordance with relevant guidelines and regulations. The animal Experiments were conducted in the Biosafety Level 2 laboratory in Henan University of Animal Husbandry and Economy, and all methods were reported in accordance with ARRIVE guidelines. All applicable international, national, and/or institutional guidelines for the care and use of animals were followed. The research protocol used in this study was reviewed and approved by the Research Ethics Committee of Henan University of Animal Husbandry and Economy.

## Results

### Clinical signs and post-mortem examinations

**Figure 1:**
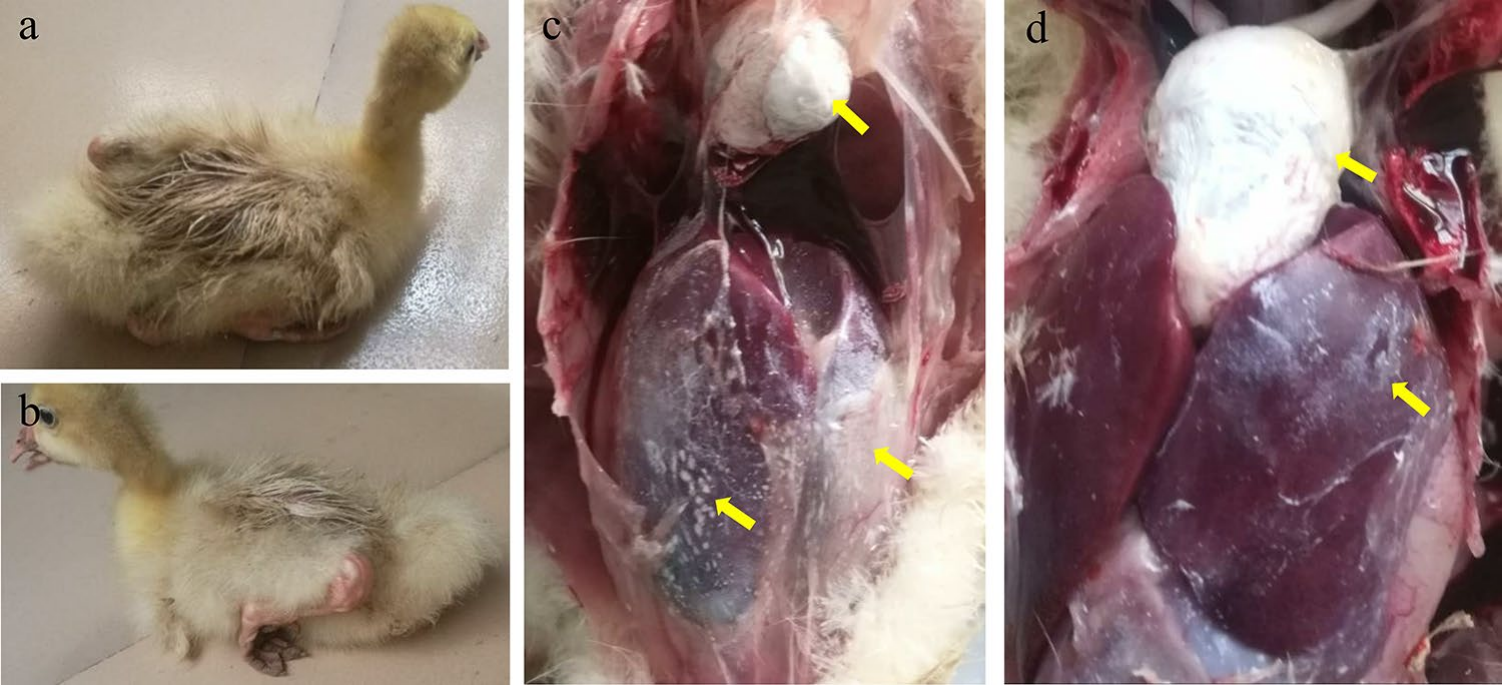
Gross lesions of diseased goslings from the commercial goose farms. (**a**, **b**) Various degrees of articular gout (**c**, **d**) Urate crystals deposition on the surfaces of the heart and liver.

For this disease outbreak, most diseased goslings were characterized by white diarrhea, anorexia, and laid on the ground (Fig. 1a,b). Among the organs collected from the diseased goslings in this outbreak, the most obvious histologic lesions were observed in liver and heart. The surfaces of the liver (Fig. 1c,d) and heart (Fig. 1c,d) exhibited acute urate deposition in all diseased goslings. In addition, the spleen and kidneys of the dead goslings also exhibited hemorrhage and swelling.

### Molecular diagnosis

**Figure 2:**
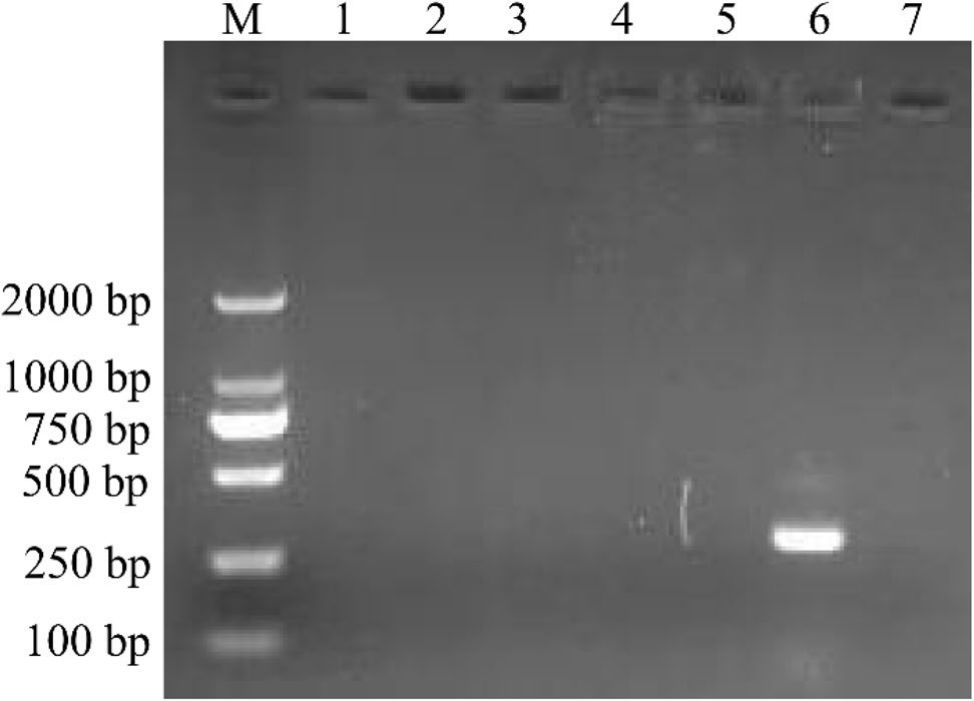
Agarose gel electrophoresis results for PCR/RT-PCR product of the potential causative agents. M, DL2000 DNA Ladder; lane 1, GRV; lane 2, TUMV; lane 3, GPV; lane 4, GHPV; lane 5, APMV-1, lane 6, GAstV; lane 7, negative control. Purified the DNA/RNA samples were used to detect the potential causative agents causing gout in gosling using PCR/RT-PCR for with specific primers, including GRV (380 bp), TUMV (401 bp), GPV (779 bp), GHPV (144 bp), APMV-1(330) and GAstV (163 bp). Original gel was presented in Supplementary Figure S3.

To identify the causative agents of the disease, PCR/RT-PCR assays were used to detect the potential causative agents. Tissue samples of the diseased goslings were confirmed to be positive for GAstV and negative for GRV, TMUV, GPV, GHPV, and APMV-1 (Fig. 2). In addition, no *Escherichia coli*, *Riemerella anatipestifer* or *Pasteurella multocida* were identified in these diseased goslings. Of these tested goslings, 100% (13/13) were positive for GAstV by RT-PCR (Supplementary Figure S2).

### Virus isolation and virus titer

**Figure 3:**
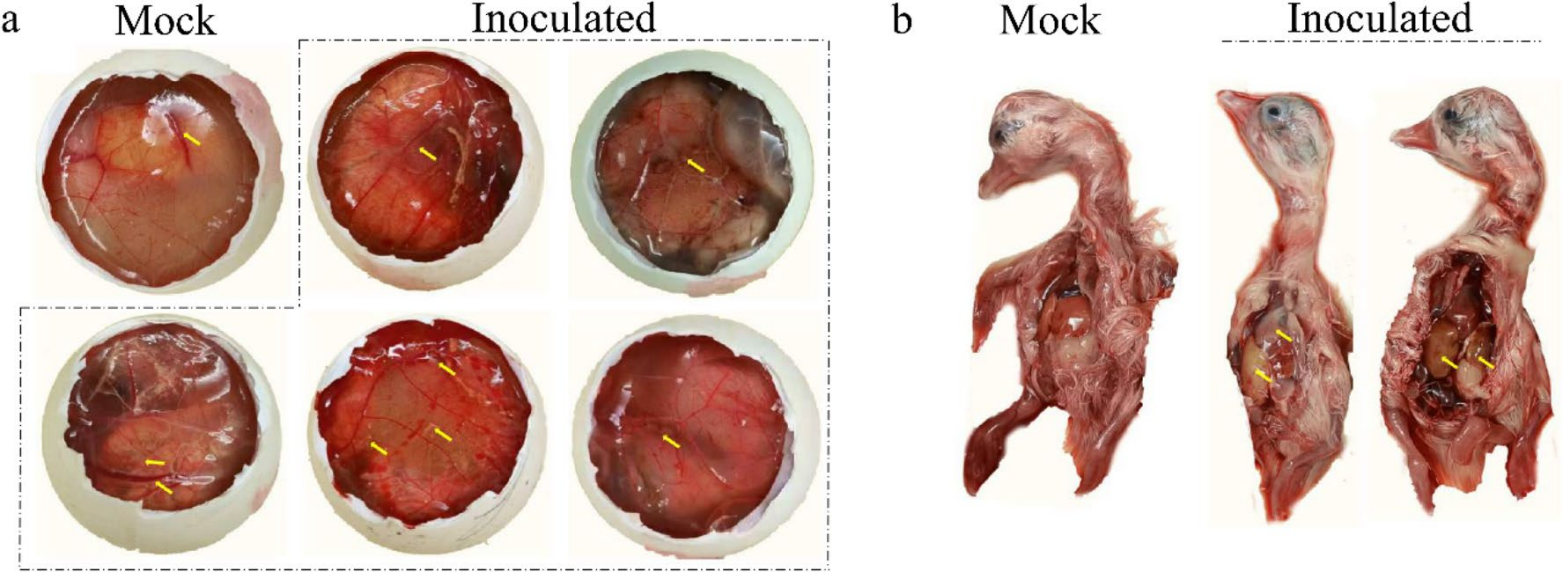
The pathogenicity of GAstV to goose embryos. (**a**) The goose embryos inoculated with fourth passage GAstV strains induced the chorioallantoic membrane increasing thicken, with urate crystals in the allantoic fluid and even depositing around great vessels and embryo bodies. (**b**) Compared to the mock embryo (**b**, left), the dead goose embryos inoculated with GAstV showed exhibited various degrees of haemorrhage and oedema (**b**, right), hepatic jaundice and liver necrosis (**b**, middle).

The homogenates of the GAstV-positive liver and kidney samples were inoculated into the chorioallantoic cavity of 15-day-old healthy goose embryos. All the goose embryos inoculated with the fourth passage of the isolated strain exhibited varying degree of hemorrhage, thickening of chorioallantoic membrane, with urate crystals in the allantoic fluids (Fig. 3a). Meanwhile, the goose embryos inoculated with the isolated strain exhibited various degrees of haemorrhage and oedema, hepatic jaundice and liver necrosis (Fig. 3b). The allantoic fluids of the goose embryos were only positive for GAstV, and GRV, TMUV, GPV, GHPV and APMV-1 were detected to be negative by PCR/RT-PCR. Eventually, a GAstV strain causing acute gosling visceral gout was isolated and named as HNZZ-4. Quantal assays showed that the infectious virus titers of the P5 and P10 passage were 10^3.7^ TCID_50_/0.1 mL and 10^4^ TCID_50_/0.1 mL, respectively.

### Whole genome sequencing and characterization

To reveal the characteristics of the newly isolated NGAstV HNZZ-4 from Henan province, central China, the whole genome was sequenced with overlapping RT-PCR and RACE strategies. Sequence analysis revealed that the complete genome sequence of NGAstV HNZZ-4 was 7172 nt in length (Accession No. ON376722). There were three open reading frames (ORFs), ORF1a (3255 nt, 14–3268), ORF1b (1551 nt, 3259–4809) and ORF2 (2115 nt, 4828–6942) in NGAstV HNZZ-4 genome. The untranslated regions (UTRs) were at the 5′ and 3′ termini of the genome sequence with length of 13 nt (5′ UTR) and 230 nt (3′ UTR, including a poly (A) tail of 24 nt).

Similar to other GAstV strains, there were several conserved motifs in ORF1a of the newly identified NGAstV HNZZ-4, including four predicted transmembrane domains (aa 231–248, aa 384–406, aa 421–443 and aa 455–477), a serine protease motif (GNSG, aa 672–675) and a nuclear localization signal motif (KKKGKTK, aa 773–779)^8,9^. By comparing with reference GAstV strains, a ribosomal frame shift signal was observed in the 10 nt overlap region between ORF1a and ORF1b of NGAstV HNZZ-4, consisting of the highly conserved ribosome frame shift sequence AAAAAAC (3259–3265) and a stop codon in ORF1a. Meanwhile, there were four conserved motifs in ORF1b of the newly identified NGAstV HNZZ-4, including DWTRYD (aa 265–270), NPSGQYSTTVDN (aa 327–339), YGDD (aa 377–380) and FGMWVK (aa 405–410). There was an 18 nt long gap between the ORF1b stop codon and the ORF2 start codon. The ORF2 gene was 2115 nt in length, encoding a capsid protein of 704 aa.

### Phylogenetic analysis

**Figure 4:**
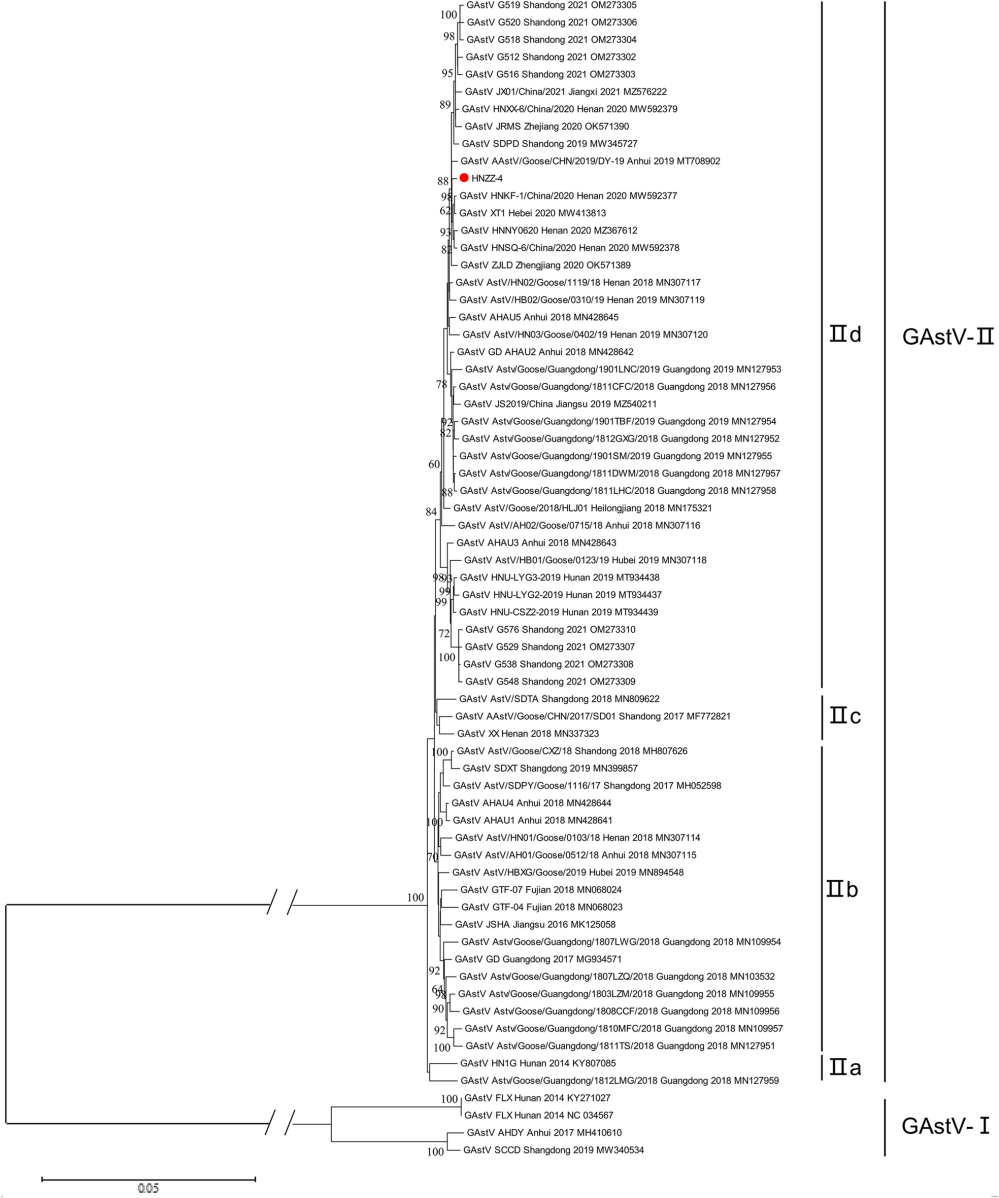
Phylogenetic analysis of complete genomes between isolated strain HNZZ-4 and AAstV reference strains. Based on the multiple alignment of complete genomes with other GAstVs and representative strains of AAstVs available from the GenBank database, phylogenetic tree was constructed with MEGA 6.0 software using the Neighbor-joining method with 1000 bootstrap replicates. Two distinct lineages: GAstV-I and GAstV-II, are indicated. The newly identified virus in this study was indicated by red solid circle.

**Figure 5:**
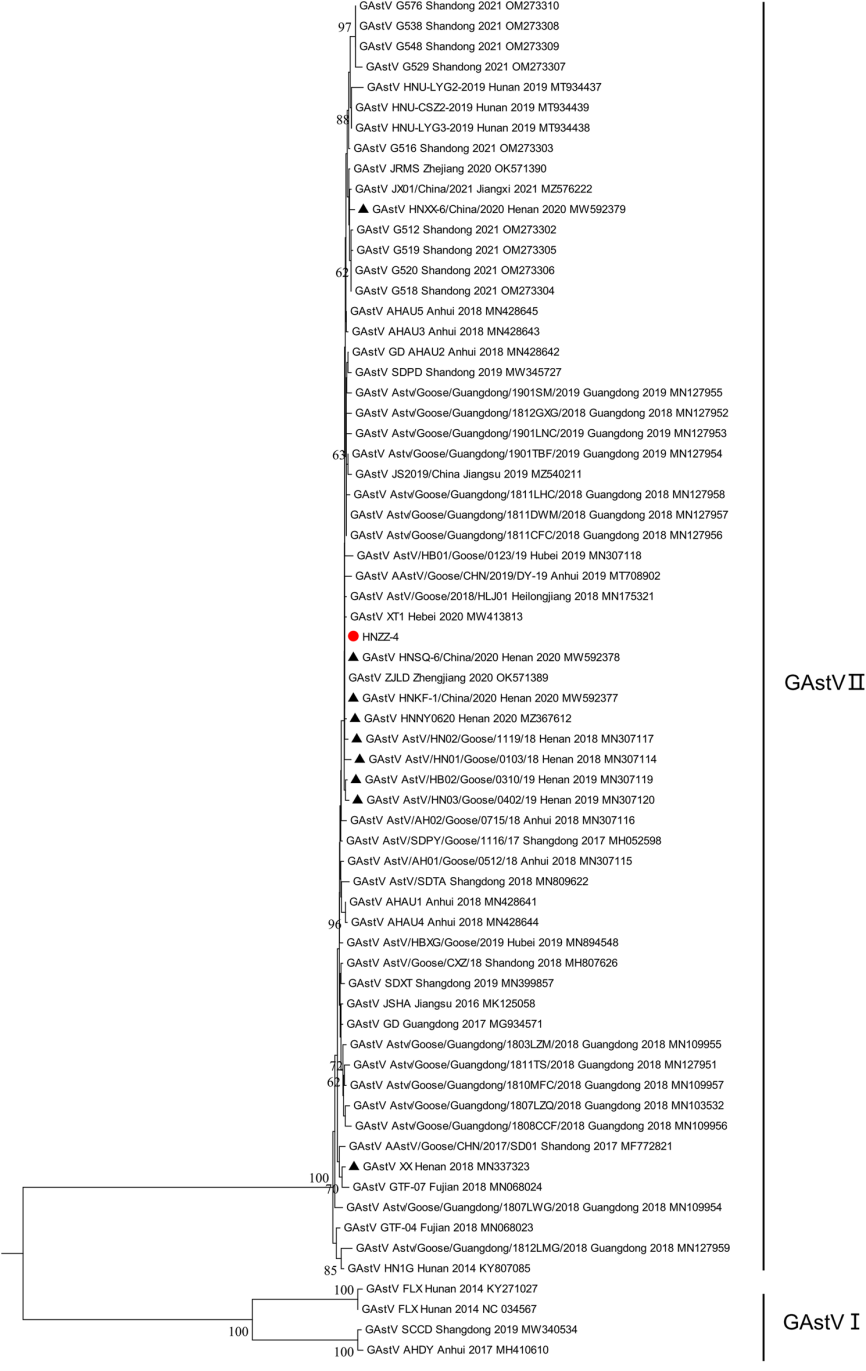
Phylogenetic analysis of GAstVs based on ORF2 encoded amino acid sequences. The newly identified virus in this study and GAstV strains isolated in Henan province were indicated by red solid circle and black triangles, respectively. Phylogenetic tree was constructed by MEGA 6.0 software using the Neighbor-joining method with 1000 bootstrap replicates.

To further explore the evolutionary characteristics of the newly identified NGAstV strain HNZZ-4 and more precisely determine the relationships among the strains circulating in different regions and periods, the phylogenetic tree of GAstVs was constructed based on the complete genome nucleotide sequences. As shown in the phylogenetic tree, GAstVs were clustered into two distinct lineages GAstV and NGAstV (Fig. 3)^5,9^. Based on previously described nomenclature method^20^, GAstV(represented by FLX) was named as GAstV-I, which comprised GAstV strains SCCD (MW340534), AHDY (MH410610), FLX (NC034567), FLX (KY271027), and closely related to DAstV-IV and TAstV-I. While NGAstV, represented by strain HN1G (KY807085) and SD01 (MF772821), was named as GAstV-II, which comprised the majority of known GAstV strains discovered after 2018 (58/59, excepting SCCD), including the newly identified NGAstV HNZZ-4. However, GAstV-II was closely related to DAstV-II and TAstV-II (Fig. 4). In addition, GAstV-II was further divided into sub-genotype IIa, IIb, IIc and IId (Fig. 4), comprising 1, 2, 15, 40 GAstV strains discovered after 2018, respectively. Indeed, the phylogenetic analysis of ORF2 encoded amino acid sequences revealed a similar grouping structure to the phylogenetic tree based on the AstVs complete genome nucleotide sequences, and more complex genetic diversity (Fig. 5).

### Genetic and amino acid sequence analysis

To further investigate the genetic characteristics of the newly identified NGAstV HNZZ-4 strain, the complete genome sequence and deduced amino acid sequence were compared with reference GAstV strains. The complete genome sequence of the HNZZ-4 with other GAstV strains in GAstV-I and GAstV-II shared identities of 57.2%–57.3% and 97.3%–99.6%, respectively. The deduced amino acid sequences of HNZZ-4 with other GAstV strains in GAstV-I and GAstV-II shared identities of 47.3%–47.5% and 97.9%–99.6% in ORF1a, 58.6%–58.9% and 98.8%–99.6% in ORF1b, 42.8%–43.7% and 96.9%–100% in ORF2, respectively.

To further explore amino acid diversity of newly identified NGAstV HNZZ-4, ORF1a, ORF1b, ORF2 encoded amino acid sequences were compared with that of reference GAstV strains. The results showed that, similar to reference GAstV strains, most of the amino acid mutations in the newly identified NGAstV HNZZ-4 were located in the 3C-like serine protease and capsid protein (Table 1), such as T428N, S555R, A782T, A224T, A/Q225R, and T289A. Meanwhile, some mutations were only found in NGAstV HNZZ-4 (S/T/Q/S926F, T/I/A982S, and S/T985F in 3C-like serine protease). In addition, one mutation (V355I) occurred in the coiled helix functional region, two mutations (V555I and H589Y) occurred in the serine protease domain, and one mutation (A782T) occurred in the nuclear localization signal region of the ORF1a protein. However, the characteristic GNSG motif of the serine protease domain of ORF1a and the conserved motif KKKGKTK in the nuclear localization signal domain showed no differences among all strains. Notably, there were three amino acid mutation sites (E456D, A464N, and L540Q) in the highly variable C-terminal region capsid domain of the newly identified NGAstV HNZZ-4. These three mutation sites were components of epitopes, indicating that these mutations might alter viral antigenicity.

**Table 1:** Amino acid sequence analysis of the individual protein among HNZZ-4 and other GAstV-II strains.

Protein	Position	HNZZ-4	Others	Location
3C-like serine protease	355	I	V/I	CH
	428	N	T/N	TM
	520	T	A/T	/
	528	T	T/I	/
	533	M	M/V	/
	535	R	R/S	/
	555	I	V/I	Pro
	782	T	T/A	NLS
	926	F	S/T/Q/S	/
	982	S	T/I/A	/
	985	F	S/T	/
	1082	G	E/G	/
RdRP	61	Y	H/Y	/
	324	I	T	/
Capsid	224	T	T/A	Cap_C
	225	R	A/Q	Cap_C
	229	P	P/Q	Cap_C
	289	A	A/T	Cap_C
	376	T	T/A	Cap_C
	379	P	P/T	Cap_C
	456	**D**	E/D	Cap_P2
	464	N	A/N	Cap_P2
	540	**Q**	Q/L	Cap_P2

CH respected the coiled helix functional region.

TM respected the third transmembrane domain; Pro respected the trypsin-like serine protease motif; NLS respected the nuclear localization signal; Cap_C respected the N-terminus of the capsid core domain; Cap_P2 respected the C-terminus of the capsid spike domain.

## Discussion

Since 2017, the sudden outbreak and wide spread of a fatal visceral gout in goslings in China has resulted in substantial economic losses to goose industry^4–8,10,13^. Worse, this disease is ever-increasing since early 2020. The disease mainly affects goslings of 5–20 days old and has a mortality of over 30%. The common gross lesion is visceral urate deposition on the surfaces of viscera, including heart, liver and kidneys, as well as in joints^7,8,11,12^. Previous studies have identified the causative agent as a NGAstV distinguished from the classical GAstV^4,5,11,12^. Although the first case of NGAstV infection in China dates back to 2014, there was no enough attention paid on it until the disease broke out in many provinces in China. Moreover, the NGAstV has cross-species transmission potential between the waterfowl and chicken, which could resulted in kidney swelling and hemorrhage in commercial ducks and chicken^21–23^. In addition, no effective prevent strategy to protect against this virus is available. Continuous monitoring of the genetic diversity of dominant GAstV strains is essential to develop targeted vaccines and appropriate medicines. In this study, we integrated analyses the characteristics of GAstV from different regions and periods.

Our findings indicated that all GAstVs strains clustered into two distinct lineages: GAstV-I (Represented by FLX) and GAstV-II (NGAstV). GAstV-I comprised only FLX, AHDY and SCCD. However, GAstV-II comprised most the vast majority of GAstVs strains isolated after 2018. Furthermore, GAstV-II further evolving into four sub-genotypes: IIa, IIb, IIc, IId. Significantly, sub-genotype IId included 68.97% (40/58) GAstV strains isolated in Shandong, Jiangxi, Zhejiang, Anhui, Henan, Hebei, Guangdong, Heilongjiang and Hunan after 2018, indicating that sub-genotype IId has become the dominant one circulating in geese flocks in China. In this study, the newly identified GAstV strain HNZZ-4 and other GAstV strains isolated in Henan province after 2018 were clustered into sub-genotype IId, suggesting that sub-genotype IId was also the dominant one in Henan province. These findings reminded us that the development of candidate vaccines and therapeutic agents should focus on strains in GAstV-II sub-genotype IId, and highlighted the importance of continuous monitoring of GAstV evolution. Although the pathogenicity, antigenicity, and virulence of GAstV-II sub-genotype IId strains are currently unknown and require further investigation, the unique molecular properties of GAstV-II sub-genotype IId strains may provide immunoprotecting.

Existing studies have demonstrated that GAstVs circulating in geese had complex genetic diversity. Due to both vertical and horizontal transmission among embryos, goslings and geese, it is difficult to prevent or control GAstV infection^7,14^. We attempted to determine the relation between geographical distance and genetic similarity of GAstVs. However, there was no clue regarding the relationships between the genotypes of current GAstVs and geography. Shandong, Henan, Jiangsu, Hebei, Zhejiang, Fujian and Guangdong are the main goose raising provinces in China. More than ten billion geese are raised in these provinces every year. Due to the backwardness mode of breeding, the absence of biosecurity protection measures and the increasing number of waterfowls, China has experienced ever-increasing infectious disease outbreaks. Furthermore, in terms of adjacent geographic location and convenient transportation, and geese goods are traded frequently between these provinces, which may have result in the random spreading of GAstVs in many provinces. Therefore, cessation of cross-regional trade of live waterfowls and embryos is crucial for prevention the spread of GAstVs between different regions. Meanwhile, the combination of an accurate detection method, strict quarantine and elimination will be an efficient way to prevention the spread of the virus.

The ORF2 of Astrovirus encodes the viral capsid polyprotein, which interacts directly with the host cells and is directly related to host spectrum and virulence. The genetic evolution tree based on the ORF2 can better demonstrate the genetic variation of GAstVs. Phylogenetic tree based on the amino acid sequences of capsid indicated that wider genetic heterogeneity of the capsid of GAstVs. The results of amino acid analysis of the individual protein of GAstVs demonstrated amino acid substitutions and site mutations occur mainly on capsid proteins. It is especially noteworthy that there were three mutation sites (E456D, A464N and L540Q) in the highly variable C-terminal region of capsid of GAstVs. The astrovirus capsid spike domain alone contains the receptor-binding site, and is responsible for attachment and entry into host cells^24^. A critical step in species specificity is the initial interaction between a virus and host cell, and tiny changes in receptor binding sites can play a key role in cross-species transmission^25^. Therefore, this may partly explains the broadening of GAstV host spectrum and the occurrence of cross-species transmission^22,23^.

GAstV causing gout in goslings in China has experienced complex evolutionary scenarios in recent years. Our results suggested that GAstV-II strains were the predominant strains, while sub-genotype IId is the current domain genotype, and continuous surveillance are needed. The evolution and transmission of GAstVs upon their emergence and spreading involves a complex, variable series of steps. Our findings improve the understanding of the genetic heterogeneity of GAstVs in China, providing a foundation for developing effective measures to protect waterfowl from GAstV infection, which would promote goose industry development in China.

## Supplementary Information

Supplementary Figure S1.

Supplementary Figure S2.

Supplementary Figure S3.

Supplementary Tables.

## Data Availability

All data generated or analyzed during this study are included in this published article [and its supplementary information files]. The genome sequence of GAstV HNZZ-4 obtained in this study were submitted to GenBank under accession number ON376722 (https://www.ncbi.nlm.nih.gov/).
