# Supplementary material for: Isolation and genomic characterization of one novel goose astrovirus causing acute gosling gout in China
Source: Sci Rep. 2023 Jun 29;13:10565. doi: 10.1038/s41598-023-37784-9 (PMC10310827; doi:10.1038/s41598-023-37784-9)
Supplement: Supplementary file 4 — Supplementary Tables. [file 41598_2023_37784_MOESM4_ESM.docx]

Table S1. Primers used in this study for detection of the potential viral pathogen

| **Primers** | **Sequences (5’-3’)** | **Amplicon size (bp)** | **Reference** |
| --- | --- | --- | --- |
| AIV-F | GGCGACTACTACCAACCCA | 435 | Yao et al., 2019 |
| AIV-R | CTGCTGTTCCTGCCGATAT |  |  |
| GRV-F | TGAGACGCCTGACTACGATT | 380 | Niu et al., 2017 |
| GRV-R | ATGCTTGGAGTGAGACGACT |  |  |
| TMUV-F | GCCACGGAATTAGCGGTTGT | 401 | Su et al., 2011 |
| TMUV-R | TAATCCTCCATCTCAGCGGTGTAG |  |  |
| GPV-F | AGACTTATCAACAACCATCAYT | 779 | Li et al., 2017 |
| GPV-R | TCACTTATTCCTGCTGTAG |  |  |
| GHPV-F | GAGGTTGTTGGAGTGACCACAATG | 144 | Zhang et al., 2018 |
| GHPV-R | ACAACCCTGCAATTCCAAGGGTTC |  |  |
| AMPV-1 F | CACCGGCAACCCTATTCTGT | 330 | Yao et al., 2019 |
| AMPV-1 R | AGTGCGCCTTCAGTCTTTGA |  |  |
| GAstV F | AAGAAGAGGCTGAGTACTGGA | 163 | Zhang et al., 2020 |
| GAstV-R | GGTGACATTATCCCTGAG |  |  |

| **Primers** | **Sequences (5’-3’)** | **Amplicon size (bp)** | **Purpose** | **Reference** |
| --- | --- | --- | --- | --- |
| GAstV 1F | CCGAAACAGCGATATGGCGG | 1097 | Genome sequencing | Jin et al., 2018 |
| GAstV 1097R | CAATCCGATACTGCACCGCAA |  |  |  |
| GAstV 973F | AGTTGAGAAGCTCATACCGC | 1199 | Genome sequencing |  |
| GAstV 2171R | CAAAATCCTTGCACTGGTCTGC |  |  |  |
| GAstV 1971F | CTGGTTGGTGTATTATTGATGGAAA | 1200 | Genome sequencing |  |
| GAstV 3170R | GACCATCAAACAATGAGTGCAT |  |  |  |
| GAstV 3044F | GAGCAGCGCAAAAGGATCTG | 1499 | Genome sequencing |  |
| GAstV 4543R | TAAGACCACAGAAAGTCATA |  |  |  |
| GAstV 4440F | GACACGAATGTCGTCATAG | 1620 | Genome sequencing | Zhang et al., 2021 |
| GAstV 6059R | GACAGTGGTACCTTGTCCAG |  |  |  |
| GAstV 5942F | CAGCAGGATTTATCAGACAG | 1059 | Genome sequencing |  |
| GAstV 7000R | TGCAGCTGTACCCTCGATCCT |  |  |  |
| GAstV 6795F | GACTTCCACCTAGCAGTCTC | NA | 3’RACE |  |
| GAstV 6838F | AAGAAGAGGCTGAGTACTGGA |  | nest PCR |  |
| GAstV 433R | GTTGGACAAGAGCTCCACATCG | NA | 5’RACE |  |
| GAstV 224R | GCATTACACTTCATGAGAGCA |  | nest PCR |  |

Table S2. Primers used for the amplification of full-length genomes of the GAstVs strains

Table S3. GAstV strains used for sequence alignment and phylogenetic analysis

| **Strain Name** | **Accession No.** | **Collection Date** | **Strain Region** |
| --- | --- | --- | --- |
| HN1G | KY807085 | 2014 | Hunan |
| FLX | NC_034567 | 2014 | Hunan |
| FLX | KY271027 | 2014 | Hunan |
| JSHA | MK125058 | 2016 | Jiangsu |
| JSCZ4 | MG882766 | 2017 | Jiangsu |
| AHCZ4 | MG882765 | 2017 | Anhui |
| AHCZ2 | MG882764 | 2017 | Anhui |
| SDPY | MH052598 | 2017 | Shandong |
| SD01 | MF772821 | 2017 | Shandong |
| GD | MG934571 | 2017 | Guangdong |
| AHDY | MH410610 | 2017 | Anhui |
| AHHF | MN099162 | 2018 | Anhui |
| 1810MFC | MN109957 | 2018 | Guangdong |
| 1808CCF | MN109956 | 2018 | Guangdong |
| 1803LZM | MN109955 | 2018 | Guangdong |
| 1807LWG | MN109954 | 2018 | Guangdong |
| 1812LMG | MN127959 | 2018 | Guangdong |
| 1811LHC | MN127958 | 2018 | Guangdong |
| 1811DWM | MN127957 | 2018 | Guangdong |
| 1811CFC | MN127956 | 2018 | Guangdong |
| 1812GXG | MN127952 | 2018 | Guangdong |
| 1807LZQ | MN127952 | 2018 | Guangdong |
| 1811TS | MN127951 | 2018 | Guangdong |
| AHAU2 | MN428642 | 2018 | Anhui |
| GTF-07 | MN068024 | 2018 | Fujian |
| HLJ01 | MN175321 | 2018 | Heilongjiang |
| XX | MN337323 | 2018 | Henan |
| CXZ | MH807626 | 2018 | Shandong |
| AH01 | MN307115 | 2018 | Anhui |
| AH02 | MN307116 | 2018 | Anhui |
| HN01 | MN307114 | 2018 | Henan |
| AHAU5 | MN428645 | 2018 | Anhui |
| AHAU4 | MN428644 | 2018 | Anhui |
| AHAU3 | MN428643 | 2018 | Anhui |
| AHAU1 | MN428641 | 2018 | Anhui |
| GTF-04 | MN068023 | 2018 | Fujian |
| SDTA | MN809622 | 2018 | Shandong |
| 1901SM | MN127955 | 2019 | Guangdong |
| 1901TBF | MN127954 | 2019 | Guangdong |
| 1901LNC | MN127953 | 2019 | Guangdong |
| SDPD | MW345727 | 2019 | Shandong |
| SCCD | MW340534 | 2019 | Sichuan |
| DY-19 | MT708902 | 2019 | Anhui |
| HNU-CSZ2-2019 | MT934439 | 2019 | Hunan |
| HNU-LYG3-2019 | MT934438 | 2019 | Hunan |
| HNU-LYG2-2019 | MT934437 | 2019 | Hunan |
| HN03 | MN307120 | 2019 | Henan |
| HB02 | MN307119 | 2019 | Hebei |
| HB01 | MN307118 | 2019 | Hebei |
| HN02 | MN307117 | 2019 | Henan |
| HBXG | MN894548 | 2019 | Hubei |
| TZ03 | MW353015 | 2019 | Jiangsu |
| JS2019 | MZ540211 | 2019 | Jiangsu |
| HNXX-6 | MW592379 | 2020 | Henan |
| HNSQ-6 | MW592378 | 2020 | Henan |
| JX01 | MZ576222 | 2020 | Jiangxi |
| HNKF-1 | MW592377 | 2020 | Henan |
| XT1 | MW413813 | 2020 | Hebei |
| G512 | OM273302 | 2021 | Shandong |
| G516 | OM273303 | 2021 | Shandong |
| G518 | OM273304 | 2021 | Shandong |
| G519 | OM273305 | 2021 | Shandong |
| G520 | OM273306 | 2021 | Shandong |
| G529 | OM273307 | 2021 | Shandong |
| G538 | OM273308 | 2021 | Shandong |
| G548 | OM273309 | 2021 | Shandong |
| G576 | OM273310 | 2021 | Shandong |
